# Editorial: Recent advances in nutrigenomics: Making strides towards precision nutrition

**DOI:** 10.3389/fgene.2022.997266

**Published:** 2022-09-20

**Authors:** Joanne B. Cole, Rosita Gabbianelli

**Affiliations:** ^1^ Diabetes Unit and Center for Genomic Medicine, Massachusetts General Hospital, Boston, MA, United States; ^2^ Programs in Metabolism and Medical and Population Genetics, The Broad Institute of MIT and Harvard, Cambridge, MA, United States; ^3^ Department of Medicine, Harvard Medical School, Boston, MA, United States; ^4^ Division of Endocrinology, Boston Children’s Hospital, Boston, MA, United States; ^5^ Unit of Molecular Biology and Nutrigenomics, School of Pharmacy, University of Camerino, Camerino, Italy

**Keywords:** nutrigenetics, clinical practice guideline (CPG), mendelian randomization, functional food, machine learning

Precision medicine brings hope and draws attention towards advances in nutrigenomics research which elucidates the synergistic roles nutrition and genetics play in human health. In this Research Topic of *Frontiers in Genetics* and *Frontiers in Nutrition* – *Nutrigenomics*, forward-thinking reviews and original research manuscripts provided by experts in the field promote cutting edge computational and statistical approaches, discover novel nutrigenetic relationships, and provide critical guidance on evaluating and implementing nutrigenetic findings in a clinical setting to improve human health.

Functional food ingredients that have a scientifically backed impact on human health and disease are in high demand with consumers looking for both more products and more information on their source, activity, and safety. Identifying and characterizing impactful bioactives is a tedious process, typically done in a wet-lab, using specialized and expensive instruments. Doherty et al. outline the enormous potential for artificial intelligence-led functional food ingredient discovery, validation, and characterization to address these challenges in a high-throughput manner. The authors argue for an artificial intelligence integrated workflow that complements traditional approaches from the start, highlighting its power in two case studies: the discovery of Asian rice as a potential anti-inflammatory food based on predicted immunomodulatory peptides and the characterization of peptide function in a hydrolysate derived from *Vicia faba* (i.e., broad beans) previously shown to prevent muscle atrophy. These approaches pave the way for future work integrating naturally occurring genetic variation in both the food compound and the human host to understand how these genetic effects may modify efficacy and implementation in precision health.

Obesity is one of the main risk factors associated with the development of non-communicable diseases (i.e., cardiovascular disease, type 2 diabetes, hypertension, cancer, etc.), worldwide. To create preventive strategies against obesity and its sequelae, there is a need to develop approaches that integrate genetics, epigenetics, environmental factors, and their complex interactions. Using data from the Framingham Heart Study (*N* = 1,573), Lee et al. combined a genome-wide and epigenome-wide scan for body mass index with 397 dietary and lifestyle factors and their interactions using the generalized multifactor dimensionality reduction method to identify significant predictors of obesity (213 SNPs, 530 differentially methylated sites [DMs], and 49 dietary and lifestyle factors), and then compared machine learning algorithms to identify the model with the best prediction accuracy in an independent sample (*N* = 394) from the same cohort. The stochastic gradient boosting model achieved an ROC = 0.72 and its top predictors included 21 SNPs and 230 DMs in genes such as *CPT1A*, *ABCG1*, *SLC7A11*, *RNF145*, and *SREBF1* and 26 dietary factors with processed meat, calcium intake, and diet soda at the top.

While it is clear that nutrition plays a role in many chronic diseases and overall human health, there is limited consistent evidence which point to *causal* relationships between specific foods, dietary patterns, and human disease. Using Mendelian randomization (MR) genetic instrumental variable analysis, Taba et al. apply a novel method in refining genetic instruments for MR by selecting SNPs which do not demonstrate mediation by common factors such as body mass index, educational attainment, and many others. They apply this approach to assess the causal effects between 40 foods and dietary patterns and 123 blood metabolites, representing some of the most likely intermediates on the path from diet to human disease. Together with their more recently published work (Pirastu et al., 2022), the team of authors shed light on the potential for nutritional MR studies to uncover causal associations between food intake and human traits, bringing us one step closer to more efficient randomized controlled-trials and a deeper mechanistic understanding of the role foods play in the human body.

While nutrigenetics offers the promise of bringing precision nutrition to reality, many findings do not surpass or even undergo rigorous evaluation of scientific validity, an imperative first step for implementation into clinical practice. In order to make assertions about scientific validity of nutrigenetic findings, current frameworks and scoring approaches need to be evaluated, updated, and recommended for more standard use. Therefore, Keathley et al. conducted a systemic review of evidence evaluation frameworks in the fields of nutrition and/or genetics as they could be applied to nutrigenetics. They used a detailed categorization matrix with factors ranging from study design to biological plausibility, defined and refined by both an expert working group and an external expert panel, to evaluate 41 existing frameworks and in the end, recommended Keathley et al. (2022); Boffetta et al. (2012) framework with minor modifications for future use. Once gene-diet interactions are scientifically validated, then clinical utility, social implications, ease and/or barriers to implementation, and many other factors need be assessed as clinical practice guidelines are developed.

Though clinical practice guidelines in nutrigenetics do not yet exist, they could be an effective tool for clinical care providers and policy makers to translate scientifically valid findings to healthcare. In a previously published article by Keathley et al. (2022), the authors use a modified GRADE framework (scoring high in the aforementioned categorization matrix) to assess the scientific validity of two key nutrigenetic findings: 1) male *APOE-E* carriers and 2) low 31-SNP nutrigenetic risk score in overweight and obese adults – both in the context of triglyceride changes in response to omega 3 fatty acids eicosapentaenoic acid (EPA) and/or docosahexaenoic acid (DHA). In the current perspective article Keathley et al., the authors apply the AGREE II approach to consider desirable and undesirable impacts, values and preferences, and resource use to develop clinical practice guidelines. The first recommendation of which is that triglyceride responsiveness to EPA/DHA omega-3 fatty acids in males can be based on *APOE* SNPs (rs429358, rs7412), however, given the link between this locus and Alzheimer’s Disease, care-providers need to comply with local regulations and patient consent while also considering ethical and legal implications of this test. The second recommendation confirms the use of previously published 31-SNP nutrigenetic risk score to evaluate TG responsive to EPA/DHA supplementation in both male and female overweight or obese adults. Their final recommendation is to not use these nutrigenetic findings for plasma lipids, lipoproteins and apolipoproteins given the current available evidence.

Within this Special Section in Nutrigenomics, we offer but a glimpse on many aspects of diet and nutrition, nutrition’s role in health and disease, and the practical implementation of bringing precision nutrition to the public.

## References

[B3] Boffetta P. Winn D. M. Ioannidis J. P. Thomas D. C. Little J. Smith G. D. (2012). Recommendations and proposed guidelines for assessing the cumulative evidence on joint effects of genes and environments on cancer occurrence in humans. Int. J. Epidemiol. 41, 686–704. 10.1093/ije/dys010 22596931 PMC3481885

[B1] Keathley J. Garneau V. Marcil V. Mutch D. M. Robitaille J. Rudkowska I. (2022). Nutrigenetics, omega-3 and plasma lipids/lipoproteins/apolipoproteins with evidence evaluation using the GRADE approach: A systematic review. BMJ open 12, e054417. 10.1136/bmjopen-2021-054417 PMC8867311 35193914

[B2] Pirastu N. McDonnell C. Grzeszkowiak E. J. Mounier N. Imamura F. Merino J. (2022). Using genetic variation to disentangle the complex relationship between food intake and health outcomes. PLoS Genet. 18, e1010162. 10.1371/journal.pgen.1010162 35653391 PMC9162356

